# Bt crops benefit natural enemies to control non-target pests

**DOI:** 10.1038/srep16636

**Published:** 2015-11-12

**Authors:** Jun-Ce Tian, Ju Yao, Li-Ping Long, Jörg Romeis, Anthony M. Shelton

**Affiliations:** 1 State Key Laboratory Breeding Base for Zhejiang Sustainable Pest and Disease Control, Institute of Plant Protection and Microbiology, Zhejiang Academy of Agricultural Sciences, Hangzhou, Zhejiang, 310021, China; 2 Department of Entomology, Cornell University/New York State Agricultural Experiment Station (NYSAES), Geneva, New York, 14456, United States of America; 3 Institute of Plant Protection, Xinjiang Academy of Agricultural Sciences, Urumqi, Xinjiang, 830091, China; 4 Rice Research Institute, Guangxi Academy of Agricultural Sciences, Nanning, Guangxi, 530007, China; 5 Agroscope, Institute for Sustainability Sciences ISS, Zürich, 8046, Switzerland

## Abstract

Crops producing insecticidal crystal (Cry) proteins from *Bacillus thuringiensis* (Bt) control important lepidopteran pests. However, pests such as aphids not susceptible to Cry proteins may require other integrated pest management (IPM) tactics, including biological control. We fed aphids on Bt and non-Bt plants and analyzed the Bt protein residue in aphids and compared the effects of Bt plants and a pyrethroid, lambda-cyhalothrin, on the performance of three natural enemies (predators: *Coleomegilla maculata* and *Eupeodes americanus*; parasitoid *Aphidius colemani*) of the green peach aphid, *Myzus persicae*. No Bt protein residues in aphids were detected and no significant differences were recorded in the performance of pyrethroid-resistant aphids that fed on Bt broccoli expressing Cry1Ab or Cry1C, or on non-Bt broccoli plants treated or not treated with the pyrethroid. This indicated the aphids were not affected by the Cry proteins or the pyrethroid, thus removing any effect of prey quality. Tri-trophic experiments demonstrated that no *C. maculata* and *E. americanus* survived consumption of pyrethroid-treated aphids and that ovipositional behavior of *A. colemani* was impaired when provided with pyrethroid-treated aphids. In contrast, natural enemies were not affected when fed aphids reared on Bt broccoli, thus demonstrating the safety of these Bt plants for IPM.

Since genetically engineered insect-resistant crops were first commercially grown in 1996, the area planted to them has expanded rapidly. In 2014, 78.8 million ha of insect-resistant crops (cotton and maize) producing insecticidal crystal (Cry) proteins derived from *Bacillus thuringiensis* Berliner (Bt) were planted in 28 countries^1^. In 2014, Bt eggplant became available in Bangladesh and 20 fields were planted with Bt eggplant^2^. In 2015, the number increased to 108 fields. Studies have reported that Bt cotton and maize have provided substantial economic benefits and reduced the use of harmful insecticides with positive implications for biological control^1^,^3^,^4^. Moreover, widespread adoption of Bt cotton and Bt maize has suppressed regional populations of the cotton bollworm, *Helicoverpa armigera* (Lepidoptera: Noctuidae), in China^5^, pink bollworm, *Pectinophora gossypiella* (Lepidoptera: Gelechiidae), in the USA^6^, and the European corn borer, *Ostrinia nubilalis* (Lepidoptera: Crambidae), in the US^7^. These reductions have not only benefited growers of Bt crops but also non-Bt farmers surrounding them who have used fewer insecticides^5^,^7^.

Although the economic benefits and reduced sprays required for Bt crops have been well documented, the potential effect of Bt crops on non-target organisms (especially natural enemies) continues to be an active area of research with ramifications for registration of Bt crops in some countries^8^. A few studies have claimed Bt crops have negative effects on important natural enemies, especially parasitoids, but far more reports have demonstrated that Bt crops do not harm natural enemies^9^,^10^,^11^. However, while many of these assessments of the potential effects of Bt crops on natural enemies only compared Bt crops to non-Bt crops, those that compared the Bt crops to insecticide treated non-Bt crops reported significant positive effects of the Bt treatment^9^,^10^. In a risk-benefit analysis of Bt crops it is important to compare alternative technologies, including commonly-used conventional insecticides which remain the dominant insect pest control strategy globally^12^.

Studies have shown that Bt crops can control target lepidopteran pests as well as, if not better than, conventional insecticides^13^. Such studies include the pyrethroid class of insecticides that is widely used against insect defoliators, and lambda-cyhalothrin is a common pyrethroid targeting lepidopteran pests. Lepidopterans are key pests on maize^14^ and cotton^15^, but these crops are also attacked by many non-lepidopteran herbivores that are not controlled by Bt proteins. For example, the green peach aphid, *Myzus persicae* (Hemiptera: Aphididae), is a cosmopolitan aphid species that causes substantial losses to field crops and horticultural crops^16^. *M. persicae* is a member of the aphid complex that attacks both cotton and maize^16^ and exhibits a capacity to rapidly develop resistance to many insecticides including pyrethroids^17^. Although key lepidopterans can be controlled by Bt proteins, often insecticide treatments are needed for non-Bt susceptible insects including aphids. However, such insecticide uses may cause outbreaks of secondary pests if they evolve resistance and/or if insecticides decimate their natural enemies. In order to keep all pests under control, a comprehensive long-term pest management program, guided by the principles and practices of integrated pest management (IPM), should be developed^18^. The United Nations Food and Agriculture Organization (FAO) defines IPM as “the careful consideration of all available pest control techniques and subsequent integration of appropriate measures that discourage the development of pest populations and keep pesticides and other interventions to levels that are economically justified and reduce or minimize risks to human health and the environment”^19^. Biological control, using natural enemies to manage pests, is one of the foundations of IPM^20^ and natural enemies should be conserved to reduce populations of primary and secondary pests.

In the present study, we compared the effects of Bt plants and lambda-cyhalothrin on the performance of three natural enemies of *M. persicae*. The natural enemies tested represented three insect orders so we could obtain a more comprehensive perspective for potential effects of these treatments on natural enemies: *Coleomegilla maculata* (Coleoptera: Coccinellidae), a predator; *Eupeodes americanus* (Diptera: Syrphidae), a predator, and *Aphidius colemani* (Hymenoptera: Braconidae), a parasitoid. These natural enemies were provided *M. persicae* that had fed on Bt broccoli plants expressing Cry1Ab or Cry1C, non-Bt broccoli plants treated with lambda-cyhalothrin and non-Bt plants that were not treated with lambda-cyhalothrin. Several life-table parameters of the natural enemies were compared to determine the potential effects of Bt plants and lambda-cyhalothrin.

## Results

### Bt protein level of Aphid fed on Bt broccoli

No Bt Cry protein was detected in aphids sampled from Cry1Ab broccoli, Cry1C broccoli or non-Bt broccoli (n = 3).

### Aphid performance

The lambda-cyhalothrin-resistant *M. persicae* showed a high level of resistance to a commercial pyrethroid product, Warrior II, containing lambda-cyhalothrin, and no significant difference in survival was found between the lambda-cyhalothrin treatment and the control treatment (Table 1). Likewise, there was no significant difference in survival of *M. persicae* that fed on Bt broccoli that expressed Cry1Ab or Cry1C or on non-Bt broccoli (Table 1). Furthermore, there were no significant differences in any other life-table parameters of *M. persicae* due to Cry1Ab or Cry1C or non-Bt broccoli treatments (Table 1). Overall, our results indicate that there were no significant differences in any of the parameters we measured and that the lambda-cyhalothrin-resistant *M. persicae* were suitable for the tri-trophic studies described below.

### Tri-trophic bioassay with *C. maculata*

When *C. maculata* fed on lambda-cyhalothrin-treated *M. persicae*, neither 1^st^ instar *C. maculata* nor 4^th^ instar *C. maculata* could reach the next stage (Table 2). When *C. maculata* fed on *M. persicae* that had fed on Cry1Ac or Cry1C broccoli or non-Bt broccoli, no significant differences in any life-table parameters were found (Table 2). These results indicate that consumption of lambda-cyhalothrin-treated *M. persicae* was harmful to the predator but that consumption of *M. persicae* that had fed on plants expressing Cry1Ac or Cry1C was not.

### Tri-trophic bioassay with *E. americanus*

When *E. americanus* fed on lambda-cyhalothrin-treated *M. persicae*, none of them reached the pupal stage (Table 3). When *E. americanus* were supplied with *M. persicae* that had fed on Cry1Ac or Cry1C broccoli or non-Bt broccoli, there were no significant differences in any life-table parameters (including survival, larval development time, pupal duration and pupa fresh weight) among the Bt broccoli treatments and the control (non-Bt broccoli) treatment (Table 3). These results indicate that consumption of lambda-cyhalothrin-treated *M. persicae* was harmful to the predator but that consumption of *M. persicae* that had fed on broccoli plants expressing Cry1Ac or Cry1C was not.

### Tri-trophic bioassay with *A. colemani*

After being parasitized by *A. colemani* for 24 h, *M. persicae* mummies formed and adults emerged after 5–8 d. No significant differences were found in the development time (including oviposition to mummy and mummy to adult), pupal survival and female sex ratio among the four treatments, but percentage of parasitism in the lambda-cyhalothrin treatment was significantly lower than those in the Cry1Ac broccoli treatment, the Cry1C broccoli treatment and the non-Bt broccoli treatment (Table 4). These results indicate that consumption of lambda-cyhalothrin-treated *M. persicae* was harmful to the parasitoid but that consumption of *M. persicae* that had fed on plants expressing Cry1Ac or Cry1C was not.

## Discussion

Bt crops (cotton and maize) effectively control key lepidopteran pests^14^,^15^. However, most cropping systems have insect complexes in which non-lepidopterans may become more problematic when broad-spectrum insecticides, such as pyrethroids, targeting the key species are eliminated. This phenomenon has been documented in both maize and cotton^21^,^22^. Outbreak of pests can also occur when the species become resistant to insecticides^15^. The phenomenon of insecticide-induced resurgence of pests has been well documented when chemical insecticides have been used as the primary pest control method^23^,^24^,^25^. In addition there are indications that herbivores that are not sensitive to Bt toxins may benefit from the decrease in resource competition^26^,^27^ or the reduced indirect, plant-mediated competition which can be mediated by secondary plant metabolites^28^.

Aphids are a common pest in many cropping systems and insecticide-induced aphid outbreaks have been reported in many field crops including cotton, cabbage, cauliflower, and soybean^25^,^29^,^30^. Suitable pest management tactics for controlling aphids are required for Bt crops, because aphids are not affected by the Bt trait^31^,^32^. Biological control, which emphasizes the preservation and enhancement of natural enemies, is a key component of IPM and represents a significant source of sustainable control if it can be integrated with other pest suppression tactics^18^,^33^. There are many predator and parasitoid species that can control aphids effectively^34^,^35^. Thus, an understanding of how biological control integrates with Bt crops and chemical insecticides is required for sustainable IPM. In the present study, three aphid natural enemies, each from a different insect order, were evaluated for their inclusion into an IPM program when subjected to Bt broccoli or a pyrethroid treatment.

Prey or host quality effects could significantly affect the performance of predators and parasitoids and lead to misinterpretation of the potential effect of Bt crops on non-target organisms including natural enemies^8^,^10^. To avoid this problem we conducted bioassays with lambda-cyhalothrin-resistant aphids that fed on Bt broccoli or non-Bt broccoli or were treated with lambda-cyhalothrin. No significant differences in any of the measured aphid life-table parameters were observed among treatments (Table 1), which indicated that the aphids were resistant to lambda-cyhalothrin and the quality of aphids as prey/hosts for the natural enemies appeared to be equal between treatments. It is not surprising that no effects of Bt broccoli on *M. persicae* life table parameters were found because the aphids were ingesting only negligible amounts of Cry proteins as indicated by the ELISA measurement, a fact that has been reported for other aphid species and Bt plants^36^.

The ladybird beetle *C. maculata* is a common and abundant predator whose larvae and adults are major predators of aphids^37^. The syrphid fly *E. americanus* is a specialized predator of aphids and is commonly found throughout North America^38^. The larvae of syrphid flies can effectively suppress populations of aphids and help lettuce growers in California produce harvestable crops^39^. There are many parasitoids of aphids and one of the most effective is the solitary endoparasitoid, *A. colemani*, which is used for biological control of *M. persicae* and *Aphis gossypii* in greenhouses through mass releasing as well as in banker plant systems^40^,^41^.

Our tri-trophic bioassays demonstrated that broccoli plants expressing Cry1Ac and Cry1C do not harm the survival, development, weight and fecundity of *C. maculata* (Table 2). Our results are consistent with other studies that evaluated the potential effect of Bt crops on ladybird beetles. No significant difference in fitness parameters of ladybird beetles have been found when they fed on aphids, spider mites, or Bt-resistant lepidopteran larvae that had fed on Bt crops expressing different Cry proteins (Cry1Ab, Cry1Ac, Cry1F, Cry2A, and Cry3Bb1)^42^,^43^,^44^,^45^,^46^. In contrast, no ladybird beetles could reach their next development stage when they fed on pyrethroid-treated aphids. Another study investigated the susceptibility to pyrethroids of seven ladybird beetle species, including *C. maculata*, and found all were highly susceptible^47^. It was not surprising that all syrphid flies were killed when they fed on pyrethroid-treated aphids (Table 3) because field investigation have shown that densities of syrphid flies are negatively impacted by pyrethroids and other insecticides^48^. In contrast, our studies appear to be the first that demonstrated that Cry1Ac and Cry1C expressing Bt plants do not harm *E. americanus*. For the parasitoid *A. colemani*, lambda-cyhalothrin significantly reduced the percentage of parasitism, but did not impact other life-table parameters including development, pupal survival and female sex ratio (Table 4). Though *A. colemani* is susceptible to many insecticides^49^, our result indicated that *A. colemani* was not exposed to a sufficiently high dose of lambda-cyhalothrin when it was applied once to aphids used as hosts, because development and pupal survival of *A. colemani* were not impacted. However, the ovipositional behavior was impaired by lambda-cyhalothrin, and this reduced the percentage of parasitism, similar to what was documented in another parasitoid in this genus^50^. As expected, Bt broccoli did not impact the performance of *A. colemani*. This result was similar to what was demonstrated with *Diadegma insulare* (Hymemnoptera: Ichneumonidae), an important endoparasitoid of *Plutella xylostella* (Lepidoptera: Plutellidae), which was not affected when exposed to Cry1C protein in the host^51^. In contrast, the same study showed that chemical insecticides significantly reduced parasitism rates of insecticide-resistant *P. xylostella*.

While our studies confirm the lack of effects of Cry1Ac and Cry1C-transgenic plants on *C. maculata*, *E. americanus* and *A. colemani*, they do not allow us to draw a conclusion about the sensitivity of those natural enemies to the Cry proteins because it is unlikely that the natural enemies were actually exposed to the Bt proteins when provided with Bt plant-fed *M. persicae*. Numerous studies have shown that aphids in general do not ingest plant-produced Cry proteins and this is likely due to the fact that the proteins are not transported in the phloem-sap on which the aphids feed^36^. Studies that have detected Cry proteins in aphid samples, including those that cannot be explained by contamination, have reported very low amounts of Bt protein in the aphids^36^. There is, however, no validated evidence that Cry1Ac has any activity in arthropods outside the order of Lepidoptera, as demonstrated from the many studies on the non-target effects of Cry1Ac-expressing Bt cotton or purified Cry1Ac protein^10^,^52^. While information on Cry1C in the published literature is scarce, recent studies revealed no effects of this protein on the ladybird beetle *Propylea japonica* (Coleoptera: Coccinellidae)^53^ and the green lacewing *Chrysoperla sinica* (Neuroptera: Chrysopidae)^54^.

Our series of studies revealed that Bt plants expressing Cry1Ac and Cry1C do not impact predators and parasitoids of aphids, but those natural enemies are harmed by a commonly-used pyrethroid insecticide. Thus, these detailed laboratory studies provide insight into field observations on the population dynamics of non-lepidopteran species on Bt plants. For example, studies have reported that Bt cotton maintains cotton aphids at a low density, whereas populations of cotton aphids explode when chemical insecticides are used for controlling *H. armigera* in conventional cotton fields^55^. Likewise field investigations have shown that several secondary pests, particularly mirid plant bugs, have become key pests in Bt cotton fields^15^,^21^. However, this phenomenon is generally thought to be primarily due to reduced insecticide use for controlling lepidopteran pests that also served to reduce populations of non-lepidopteran insects, such as aphids^15^. Regardless, our results demonstrate that using Bt plants expressing Cry1Ac and Cry1C to control Lepidoptera does not harm this diverse set of natural enemies, while use of a common insecticide negates the biological control services they could otherwise provide. Our results contribute to the body of knowledge on Cry1 proteins from Bt that are expressed in commercialized Bt crops for control of lepidoteran pests (Cry1Ab/Cry1F for maize and Cry1Ac for cotton). Currently, there is no validated evidence that those proteins cause direct toxic effects to arthropods outside the target order of Lepidoptera^8^,^10^,^52^,^56^,^57^.

## Methods

### Plants

Two lines of transgenic broccoli (*Brassica oleracea* L., var. ‘*italica*’ ‘Green Comet’), which produces high levels of Cry1Ac (ca. 10.15 μg Cry1Ac/g fresh leaf tissue^58^) or Cry1C (1.09–1.12 μg/g fresh leaf tissue^59^), were used in this study. The expression of these proteins in Bt broccoli was verified by screening them using Bt-susceptible diamondback moth, *P. xylostella*. Non-Bt broccoli (Packman F1 Hybrid) (Harris^®^ Seeds, Rochester, NY), a similar variety of broccoli, was used as a control since ‘Green Comet’ is no longer available. Plants were grown in 6 L plastic pots in the same greenhouse at 17 ± 2 °C under a light and dark regime of 16:8 h.

Approximately 6 g of Osmocote Plus patterned release fertilizer (Scotts, Marysville, OH) was placed in each pot and 500 ml of Power-Gro liquid fertilizer (Wilson Laboratories Inc., Dundas, ON, Canada) was applied weekly. 4 to 5 week-old broccolis were used in the experiments.

### Insects

A pyrethroid-resistant *M. persicae* strain was collected from a green bean field (initial > 100 individuals) at Cornell’s New York State Agricultural Experiment Station (NYSAES) in Geneva, NY in 2012 and was maintained on non-Bt broccoli (Packman) at 21 ± 2 °C under a light and dark regime of 16:8 h. For the insecticide treatment we used the pyrethroid lambda-cyhalothrin formulated as Warrior II (Syngenta, Greensboro NC). 100 ppm of lambda-cyhalothrin was applied every month to select and maintain resistance for more than 10 generations. Part of the strain was allowed to settle on Cry1Ac broccoli and Cry1C broccoli for 3–5 generations before being used in tri-trophic bioassay with *C. maculata, E. americanus* and *A. colemani*.

An insecticide-susceptible *M. persicae* strain that was originally obtained from a greenhouse (initial > 100 individuals) at Cornell/NYSAES was reared on non-Bt broccoli, as described above, but without being sprayed with lambda-cyhalothrin. This strain was only used to maintain *E. americanus* and *A. colemani*.

*C. maculata* reared on artificial diet were used in the tests^60^. This colony originated from Pioneer Hi-Bred International, Inc. (Johnston, IA) and was maintained in a climatic chamber at 27 ± 1 °C, 50 ± 10% RH, and 16:8 h photoperiod. Newly hatched 1^st^ instar larvae were used.

A colony of *E. americanus* was originally collected from a greenhouse at Cornell/NYSAES and was reared on insecticide-susceptible *M. persicae* with non-Bt broccoli at 21 ± 2 °C under a light and dark regime of 16:8 h. Newly hatched 1^st^ instar larvae were used in bioassays.

*A. colemani* mummies were obtained from IPM Laboratory Inc. (Locke, NY) and were subsequently maintained on insecticide-susceptible *M. persicae* with non-Bt broccoli at 21 ± 2 °C under a light and dark regime of 16:8 h. Newly hatched adults were allowed to mate and feed on a sugar solution for 2 d before they were used in the bioassays.

### Bt protein level of Aphids fed on Bt broccoli

Aphids (mix stage) were collected from those settled on Cry1Ac broccoli, Cry1C broccoli or non-Bt broccoli. Each population was sampled for three replications (ca 30 mg per replication). The Bt protein concentrations in the aphid samples were determined by ELISA using Cry1Ac and Cry1C detection kits from EnviroLogix (Portland, ME). Prior to analysis, aphids were washed with PBST buffer (137 mM NaCl, 2.7 mM KCl, 10 mM Na_2_HPO_4_, 2 mM KH_2_PO_4_, 0.05% Tween-20, pH 7.4) four times to remove any Bt protein from the surface. Samples were diluted at a rate of 1:10 (mg sample: μl PBST buffer) in 1.5 ml centrifuge tubes, and ground by hand using a plastic pestle. ELISA was performed according to the manufacturer’s instructions.

### Aphid performance

Approximately 60 reproductive pyrethroid-resistant *M. persicae* were allowed to settle on non-Bt broccoli and give birth to nymphs (F_1_). After 6 h, 2–3 newborn nymphs were transferred and confined in a clip cage (diam 3 cm, ht 4 cm) on the lower leaf surface of Cry1Ac broccoli, Cry1C broccoli and non-Bt broccoli to ensure that one nymph settled per clip cage. For each treatment, five plants (replications) were used on each of which five individual aphids were investigated in separate clip cages. At the bottom of the clip cage was a hole covered with fine-mesh netting for ventilation. After 2 d, surplus nymphs were removed randomly and only a single aphid remained for monitoring the treatment. Every morning and evening, aphids were checked and mortalities were recorded. After reaching the 3^rd^ instar stage, aphids on Cry1Ac broccoli, Cry1C broccoli and non-Bt broccoli were treated with a 0.1% Bond-spreader sticker (Loveland Industry, Loveland CO) solution and another group of aphids on non-Bt broccoli was treated with a 100 ppm lambda-cyhalothrin formulation mixed with a 0.1% Bond-spreader sticker solution. The solution was applied by a hand-sprayer onto the clip-cage area. For this procedure, the clip-cage was removed and the area, including the aphid, was sprayed with ca. 1 ml of the solution. Subsequently the aphid was enclosed again in the clip-cage. After reaching adulthood, the F_2_ nymphs were counted and removed daily. The following life-table parameters were obtained: Survival (S); generation time (D); number of nymphs produced during a time span equal to D (FD); daily fecundity (daily average number of nymphs produced during the reproductive period observed, DF). Prior to the analyses, data for the individual aphids from the same plant were pooled to avoid pseudo-replications resulting in n = 5. The bioassay was carried out in a chamber at 21 ± 2 °C under a light and dark regime of 16:8 h.

### Tri-trophic bioassay with *C. maculata*

Newly hatched 1^st^ instar *C. maculata* were individually kept in a 30-ml cups and supplied with Cry1Ac broccoli-fed *M. persicae*, Cry1C broccoli-fed *M. persicae*, non-Bt broccoli-fed *M. persicae* and lambda-cyhalothrin-treated *M. persicae*. Aphids and plants in the non- lambda-cyhalothrin treatments were treated with ca. 100 ml 0.1% Bond-spreader sticker solution, while those in lambda-cyhalothrin treatments were treated with ca. 100 ml 100 ppm lambda-cyhalothrin. Aphids (mixed stages, 3–7 d after being treated with a 0.1% Bond-spreader sticker solution or 100 ppm lambda-cyhalothrin) were supplied (transferred into cups) daily and always available *ad libitum*. A piece of untreated broccoli leaf and a water-saturated cotton ball was also provided on the bottom of each cup to maintain humidity. *C. maculata* were checked every morning and evening, and the following parameters were recorded: survival and developmental time of larvae and pupae. In addition, newly emerged *C. maculata* adults were weighed. The experiment was initiated with 30 *C. maculata* larvae for each treatment.

For assessing fecundity, 10 pairs of newly emerged *C. maculata* adults from each treatment were kept in individual Petri dishes (diam 9 cm) and allowed to mate. Adults were fed shrimp eggs and agar solution for 20 d, according to the procedures of Li *et al.*^60^. Eggs of *C. maculata* were removed and recorded daily. To investigate egg-hatching rates, 30 egg masses (3 masses from each of 10 pairs) from each treatment were randomly selected and put into individual Petri dishes (diam 9 cm) and monitored until eggs hatched.

Since all 1^st^ instar *C. maculata* were dead when they were supplied with lambda-cyhalothrin-treated *M. persicae*, an additional treatment was added. A group of 30 1^st^ instar *C. maculata* was supplied with non-Bt broccoli-fed *M. persicae*. After reaching the 4^th^ instar, lambda-cyhalothrin-treated *M. persicae* were provided to them to evaluate the performance of *C. maculata* as described above.

### Tri-trophic bioassay with *E. americanus*

Newly hatched 1^st^ instar *E. americanus* were individually kept in a 30-ml cups and supplied with Cry1Ac broccoli-fed *M. persicae*, Cry1C broccoli-fed *M. persicae*, non-Bt broccoli-fed *M. persicae* and lambda-cyhalothrin-treated *M. persicae*. Aphids and plants in the non-lambda-cyhalothrin treatments were treated with ca. 100 ml 0.1% Bond-spreader sticker solution, while those in lambda-cyhalothrin treatments were treated with ca. 100 ml 100 ppm lambda-cyhalothrin. Aphids (mixed stages, 3–7 d after being treated with 0.1% Bond-spreader sticker solution or 100 ppm lambda-cyhalothrin) were supplied (transferred into cups) daily and always available *ad libitum*. A piece of untreated broccoli leaf and a water-saturated cotton ball were also provided on the bottom of each cup to maintain humidity. *E. americanus* were assessed every morning and evening, and the following parameters were recorded: survival and developmental time of larvae and pupae and the pupal fresh weight. The experiment was initiated with 20 *E. americanus* larvae for each treatment.

### Tri-trophic bioassay with *A. colemani*

30 reproductive pyrethroid-resistant *M. persicae* were allowed to settle on a new Cry1Ac broccoli, Cry1C broccoli and non-Bt broccoli leaf and give birth to nymphs. After 6 h, *M. persicae* adults were removed and only newborn nymphs were kept. After newborn nymphs reached the 3^rd^ instar, 13 *M. persicae* were transferred and confined in a clip cage on each of Cry1Ac broccoli, Cry1C broccoli and non-Bt broccoli. After 6 h, *M. persicae* on Cry1Ac broccoli, Cry1C broccoli and non-Bt broccoli were applied with a 0.1% Bond-spreader sticker solution and another group of *M. persicae* on non-Bt broccoli was treated with a solution of 100 ppm lambda-cyhalothrin and 0.1% Bond-spreader sticker. The solution was applied by hand-sprayer onto the clip cage area including the aphids as in the aphid performance experiment described above. In total ca 1 ml was used per clip cage area. After 24 h, surplus nymphs in clip cages were removed randomly and only 10 aphids in each cup were kept. A mated, 2-d old *A. colemani* female was introduced into each cage for a 24-h ovipositional period. Aphids were checked for the presence of mummies (indicating parasitism) daily. Mummies were transferred into a 30-ml cup individually and monitored daily for adult parasitoid emergence. The following life-table parameters were assessed: development time (including oviposition to mummy and mummy to adult), percentage of parasitism, pupal survival and female sex ratio. In each treatment, five replications (5 plants with one cage each) were applied.

### Statistical analyses

Survival analysis of *C. maculata* and *E. americanus* was conducted using the Wilcoxon test for homogeneity. Data on other life table parameters of tested insects were analyzed using one-way ANOVA and Tukey’s multiple comparison tests. Before analysis, all percentage data were arcsine transformed, but untransformed means are presented. All statistical calculations were performed with SAS version 9.1 package.

## Additional Information

**How to cite this article**: Tian, J.-C. *et al.* Bt crops benefit natural enemies to control non-target pests. *Sci. Rep.* **5**, 16636; doi: 10.1038/srep16636 (2015).

## Figures and Tables

**Table 1:** Performance of *Myzus persicae* on Cry1Ac broccoli, Cry1C broccoli, non-Bt broccoli and lambda-cyhalothrin treatments.

Parameter^a^	Cry1Ac Broccoli	Cry1C Broccoli	Non-Bt Broccoli	Lambda-cyhalothrin^b^	Statistics^c^
S	92.0 ± 4.9	96.0 ± 4.0	92.0 ± 4.9	88.0 ± 4.9	*F* = 0.49; *df* = 3, 19; *P* = 0.70
D	9.3 ± 0.2	9.3 ± 0.2	9.2 ± 0.1	9.4 ± 0.3	*F* = 0.18; *df* = 3, 19; *P* = 0.91
FD	36.6 ± 1.9	34.9 ± 1.5	37.0 ± 1.2	36.2 ± 1.7	*F* = 0.32; *df* = 3, 19; *P* = 0.81
DF	4.0 ± 0.3	3.8 ± 0.3	4.1 ± 0.2	4.0 ± 0.2	*F* = 0.28; *df* = 3, 19; *P* = 0.84

For each treatment, five plants (replications) were used on each of which five individual aphids (clip cages) were investigated.

^a^S: survival (%); D: generation time (days); FD: number of nymphs produced during a time span when F_2_ start to give birth; DF: daily fecundity.

^b^*M. persicae* were treated with a 100 ppm lambda-cyhalothrin when they reached the 3^rd^ instar stage.

^c^No significant difference was detected among treatments (One-way ANOVA, P < 0.05).

**Table 2:** Life-table parameters of *Coleomegilla maculata* fed with *M. persicae* that fed on Cry1Ac broccoli, Cry1C broccoli, non-Bt broccoli and lambda-cyhalothrin-treated broccoli.

Parameters	Cry1Ac Broccoli	Cry1C Broccoli	Non-Bt Broccoli	Lambda-cyhalothrin	Lambda-cyhalothrin^a^	Statistics
^†^Survival (%)	93.3 a	93.3 a	93.3 a	0 b	0 b	*χ*^*2*^ = 150.9; *df* = 4; *P* < 0.001
^*^Development time (days)						
1^st^ instar stage	2.1 ± 0.1 (30)	2.2 ± 0.1 (28)	2.3 ± 0.1 (29)	—	/	*F* = 1.48; *df* = 2, 86; *P* = 0.23
2^nd^ instar stage	1.9 ± 0.1 (30)	1.9 ± 0.1 (28)	1.9 ± 0.1 (28)	—	/	*F* = 0.02; *df* = 2, 85; *P* = 0.98
3^rd^ instar stage	2.0 ± 0.1 (30)	2.1 ± 0.1 (28)	2.1 ± 0.1 (28)	—	/	*F* = 2.51; *df* = 2, 85; *P* = 0.09
4^th^ instar stage	4.0 ± 0.1 (28)	4.0 ± 0.1 (28)	4.1 ± 0.1 (28)	—	—	*F* = 0.09; *df* = 2, 83; *P* = 0.96
Pupal stage	3.4 ± 0.1 (28)	3.3 ± 0.01 (28)	3.3 ± 0.1 (28)	—	—	*F* = 0.34; *df* = 2, 83; *P* = 0.71
Larvae-adults	13.3 ± 0.1 (28)	13.5 ± 0.1 (28)	13.6 ± 0.1 (28)	—	—	*F* = 2.20; *df* = 2, 83; *P* = 0.12
*Female fresh weight (mg)	11.8 ± 0.3 (15)	11.8 ± 0.2 (13)	12.2 ± 0.2 (17)	—	—	*F* = 1.11; *df* = 2, 44; *P* = 0.34
*Male fresh weight (mg)	9.8 ± 0.2 (13)	9.2 ± 0.2 (15)	9.8 ± 0.3 (11)	—	—	*F* = 1.65; *df* = 2, 38; *P* = 0.21
*Total fecundity	70.0 ± 10.4 (10)	78.8 ± 19.3 (10)	75.4 ± 15.8 (10)	—	—	*F* = 0.08; *df* = 2, 29; *P* = 0.93
*Egg hatching rate (%)	70.7 ± 4.3 (30)	76.4 ± 2.2 (30)	72.6 ± 4.2 (30)	—	—	*F* = 0.66; *df* = 2, 89; *P* = 0.52

The experiment started with 30 larvae in each treatment. Number of replicates is given in parentheses.

/signifies data were not recorded.

^†^Wilcoxon test (*P* < 0.05). Means followed by different letters are significantly different.

*One-way ANOVA (*P* < 0.05).

-signifies no *C. maculata* reached next stage, no data collected.

^a^Lambda-cyhalothrin-treated *M. persicae* were supplied to *C. maculata* when they reached 4^th^ instar after feeding non-Bt broccoli-fed *M. persicae*.

**Table 3:** Life-table parameters of *Eupeodes americanus* fed with *M. persicae* that fed on Cry1Ac broccoli, Cry1C broccoli, non-Bt broccoli and lambda-cyhalothrin-treated broccoli.

Parameters	Cry1Ac Broccoli	Cry1C Broccoli	Non-Bt Broccoli	Lambda-cyhalothrin	Statistics
^†^Survival (%)	95.0 a	90.0 a	95.0 a	0 b	*χ*^*2*^ = 78.1; *df* = 3; *P* < 0.01
*Larval development time (days)	7.9 ± 0.3 (19)	8.1 ± 0.3 (18)	7.6 ± 0.3 (19)	—	*F* = 0.79; *df* = 2, 55; *P* = 0.46
*Pupal duration (days)	5.8 ± 0.2 (19)	5.8 ± 0.2 (18)	6.0 ± 0.2 (19)	—	*F* = 0.24; *df* = 2, 55; *P* = 0.79
*Larval to adult (days)	13.7 ± 0.3 (19)	14.0 ± 0.3 (18)	13.6 ± 0.4 (19)	—	*F* = 0.44; *df* = 2, 55; *P* = 0.65
*Pupa fresh weight (mg)	20.1 ± 0.8 (19)	19.2 ± 0.8 (18)	19.7 ± 0.9 (19)	—	*F* = 0.30; *df* = 2, 55; *P* = 0.74

The experiment started with 20 larvae in each treatment. Number of replicates is given in parentheses.

^†^Wilcoxon test (*P* < 0.05). Means followed by different letters are significantly different.

*One-way ANOVA (*P* < 0.05).

-signifies no *Eupeodes americanus* survived, no data collected.

**Table 4:** Life-table parameters of *Aphidius colemani* parasitized with *M. persicae* that fed on Cry1Ac broccoli, Cry1C broccoli, non-Bt broccoli and lambda-cyhalothrin-treated broccoli.

Parameters	Cry1Ac Broccoli	Cry1C Broccoli	Non-Bt Broccoli	Lambda-cyhalothrin	Statistics
Ovipositon to mummy (days)	8.9 ± 0.2 a	8.9 ± 0.1 a	9.1 ± 0.2 a	9.2 ± 0.1 a	*F* = 0.67; *df* = 3, 19; *P* = 0.58
Mummy to adult (days)	6.3 ± 0.1 a	6.3 ± 0.1 a	6.4 ± 0.1 a	6.0 ± 0.1 a	*F* = 2.60; *df* = 3, 19; *P* = 0.09
Ovipostion to adult (days)	15.3 ± 0.2 a	15.2 ± 0.2 a	15.5 ± 0.2 a	15.2 ± 0.1 a	*F* = 0.74; *df* = 3, 19; *P* = 0.54
Percentage of parasitism (%)	75.6 ± 3.9 a	78.8 ± 3.2 a	83.8 ± 2.3 a	54.0 ± 2.5 b	*F* = 14.7; *df* = 3, 19; *P* < 0.01
Pupal survival (%)	75.4 ± 3.2 a	79.4 ± 3.6 a	86.6 ± 2.2 a	71.4 ± 5.9 a	*F* = 2.67; *df* = 3, 19; *P* = 0.08
Female sex ratio (%)	58.8 ± 2.8 a	64.4 ± 2.6 a	62.6 ± 3.7 a	58.4 ± 5.3 a	*F* = 0.62; *df* = 3, 19; *P* = 0.61

Means ( ± SE) within a row followed by different letters are significantly different (One-way ANOVA, *P* < 0.05); N = 5.
